# Effects of alternative splicing events and transcriptome changes on kidney stone formation

**DOI:** 10.1007/s00240-021-01293-z

**Published:** 2022-01-08

**Authors:** Qunsheng Yan, Yang Chen, Haoran Liu, Guoxiang Li, Chaozhao Liang, Zongyao Hao

**Affiliations:** 1 Department of Urology, The First Affiliated Hospital of Anhui Medical University, Hefei, China; 2 Institute of Urology, Anhui Medical University, Hefei, China; 3 Anhui Province Key Laboratory of Genitourinary Diseases, Anhui Medical University, Hefei, China

**Keywords:** RNA-seq, Kidney stone, Nephrocalcinosis, Alternative splicing

## Abstract

During the development of urinary stone disease, the formation of tiny crystals that adhere to the renal tubular epithelium induces epithelial cell damage. This damage and repair of the epithelium is associated with the establishment of more crystal adhesion sites, which in turn stimulates further crystal adhesion and, eventually, stone formation. Deposited crystals typically cause changes in epithelial cell gene expression, such as transcriptome changes and alternative splicing events. Although considered important for regulating gene expression, alternative splicing has not been reported in studies related to kidney stones. To date, whether alternative splicing events are involved in the regulation of stone formation and whether crystallographic cell interactions are regulated by alternative splicing at the transcriptional level have remained unknown. Therefore, we conducted RNA sequencing and alternative splicing-related bioassays by modeling the in vitro stone environment. Many alternative splicing events were associated with crystallographic cell interactions. Moreover, these events regulated transcription and significantly affected the capacity of crystals to adhere to renal tubular epithelial cells and regulate apoptosis.

## Introduction

The incidence of renal stone disease has more than doubled in the past 40 years, with between 7 and 11% of the population at risk of kidney stone-related symptoms each year [1]. The formation of urinary stones involves several microscopic processes, such as crystal formation, adhesion and growth [2]. During these processes, renal tubular epithelial cells closely interact with adherent crystals, which affect cell adhesion, apoptosis, and inflammatory responses [3–5]. These interactions play important roles in the eventual formation of stones.

Alternative splicing events (ASEs) have been widely reported to be of importance for the regulation of gene expression, but related results exhibit some differences in kidney stones and kidney stone-related injury. Previously, the specific impact of selective splicing associated with damage caused by crystal deposition was unknown; however, we carried out relevant RNA sequencing (RNA-seq) analyses, focusing on splicing. We used calcium oxalate nodule nanocrystals to treat renal tubular epithelial cells, after harvesting the cells, performed high-throughput sequencing. The aim was to discover associations between differential ASEs occurring at the transcriptional level and differentially expressed genes (DEGs). The analysis revealed an alternative splicing (AS) regulation mechanism during crystal–cell interactions. We identified many ASEs and their important roles in CaOx-induced anomalous transcriptional regulation, particularly related to cell adhesion and apoptosis.

## Methods

### Modeling and RNA sequencing

We used calcium oxalate stones previously collected in the clinic to obtain calcium oxalate stone nanocrystals by a process comprising ball milling and pulverization [6]. Calcium oxalate nanocrystals were subsequently cultured with renal tubular epithelial cells at a concentration of 100 µg/ml for 24 h (3 replicates) to establish a treatment group. Renal tubular epithelial cells grown normally for 24 h (3 replicates) were used as the control group. HK-2 cells were purchased from Nanjing Institute of Biology, Chinese Academy of Sciences, and cultured in Dulbecco's modified Eagle medium–nutrient mixture F-12 (DMEM-F12) with 10% fetal bovine serum in an incubator at 37 °C with 5% CO_2_. The RNA-seq data obtained from the treatment and control groups were evaluated to identify ASEs and DEGs. Moreover, functional clustering analyses (Gene Ontology [GO] and Kyoto Encyclopedia of Genes and Genomes [KEGG]) of DEGs were performed.

### Read alignment and differentially expressed gene analysis

Clean reads were aligned to Genome Reference Consortium Human Build 38 (GRch38) by TopHat2 [7], which revealed 4 mismatches. Uniquely mapped reads were ultimately used to calculate the read number and reads per kilobase of exons per million fragments mapped (RPKM) for each gene. The expression levels of DEGs were assessed based on RPKM. EdgeR software [8], specifically designed for the analysis of differential gene expression, was applied to screen the RNA-seq data for DEGs. The results were analyzed using fold change (FC) ≥ 2 or ≤ 0.5 and false discovery rate (FDR) ≤ 0.05 criteria to identify DEGs.

### Validation of differentially expressed genes by RT-qPCR

The specificity of the primers was verified by Prime-Blast, which is a tool designed for identifying significantly differentially expressed RNAs. Total RNA was extracted from HK-2 cells and control cells treated with calcium oxalate stone nanocrystals. The concentration and purity of RNA were found to meet the established requirements. A Takara-series RT-qPCR kit was used for reverse transcription and quantitative amplification. The cycle threshold (CT) values were corrected by the -ΔΔ CT method. Subsequently, relative RNA expression and significant differences in expression were calculated (*P* < 0.05).

### Alternative splicing analysis

Inter-sample alternative splicing events and regulated alternative splicing events (RASEs) were identified and quantitated using the ABL pipeline, as described previously [9, 10]. In brief, the ABL pipeline enables the detection of ten types of ASEs based on splice junction reads, namely exon skipping (ES), alternative 5' splice site (A5SS), alternative 3' splice site (A3SS), intron retention (IR), mutually exclusive exon (MXE), mutually exclusive 5' untranslated region (UTR; 5pMXE), mutually exclusive 3' UTR (3pMXE), cassette exon, A3SS&ES and A5SS&ES ASEs. For sample pair comparisons, Fisher’s exact test was used to determine statistical significance based on the alternative reads and model reads of the samples used as input data. We calculated the change ratio of alternatively spliced reads and constitutively spliced reads between compared samples, which was defined as the RASE ratio. A RASE ratio ≥ 0.2 and *p* value ≤ 0.05 were set as thresholds for RASE detection. For comparisons of repeated analyses, Student’s *t* test was used to evaluate significant changes in the RASE ratio. Events that were found to be significant at the *p* value cut-off of 0.05 were considered RASEs.

### Functional enrichment analysis

To sort DEGs, Gene Ontology (GO) terms and KEGG pathways into functional categories, the KEGG Ortholog-Based Annotation System (KOBAS) 2.0 server was used [11]. Hypergeometric tests and Benjamini–Hochberg-based false discovery rate (FDR) control procedures were used to determine the enrichment of each term. Reactome (http://reactome.org) pathway profiling was also performed in the functional enrichment analysis of selected gene sets.

### Other statistical analyses

Principal component analysis (PCA) was performed with the R package factoextra (https://cloud.r-project.org/package=factoextra), which shows the clustering of samples with the first two components. After normalizing the reads of each gene in the samples by tags per million (TPM), an in-house script (Sogen) was used to visualize next-generation sequence data and genomic annotations. The pheat map package in R was used to perform clustering (https://cran.r-project.org/web/packages/pheatmap/index.html) based on Euclidean distances. Student’s *t* test was used to compare two groups. We used the global test [12, 13] to determine the association of the normalized gene expression levels of RNA-binding proteins (RBPs) in different samples with different phenotypes.

## Results

### Crystal stimulation leads to significant alterations in gene expression

Analysis of DEGs in calcium oxalate stone nanocrystal-treated renal tubular epithelial cells compared with control cells revealed significant alterations in gene expression.

**Fig. 1:**
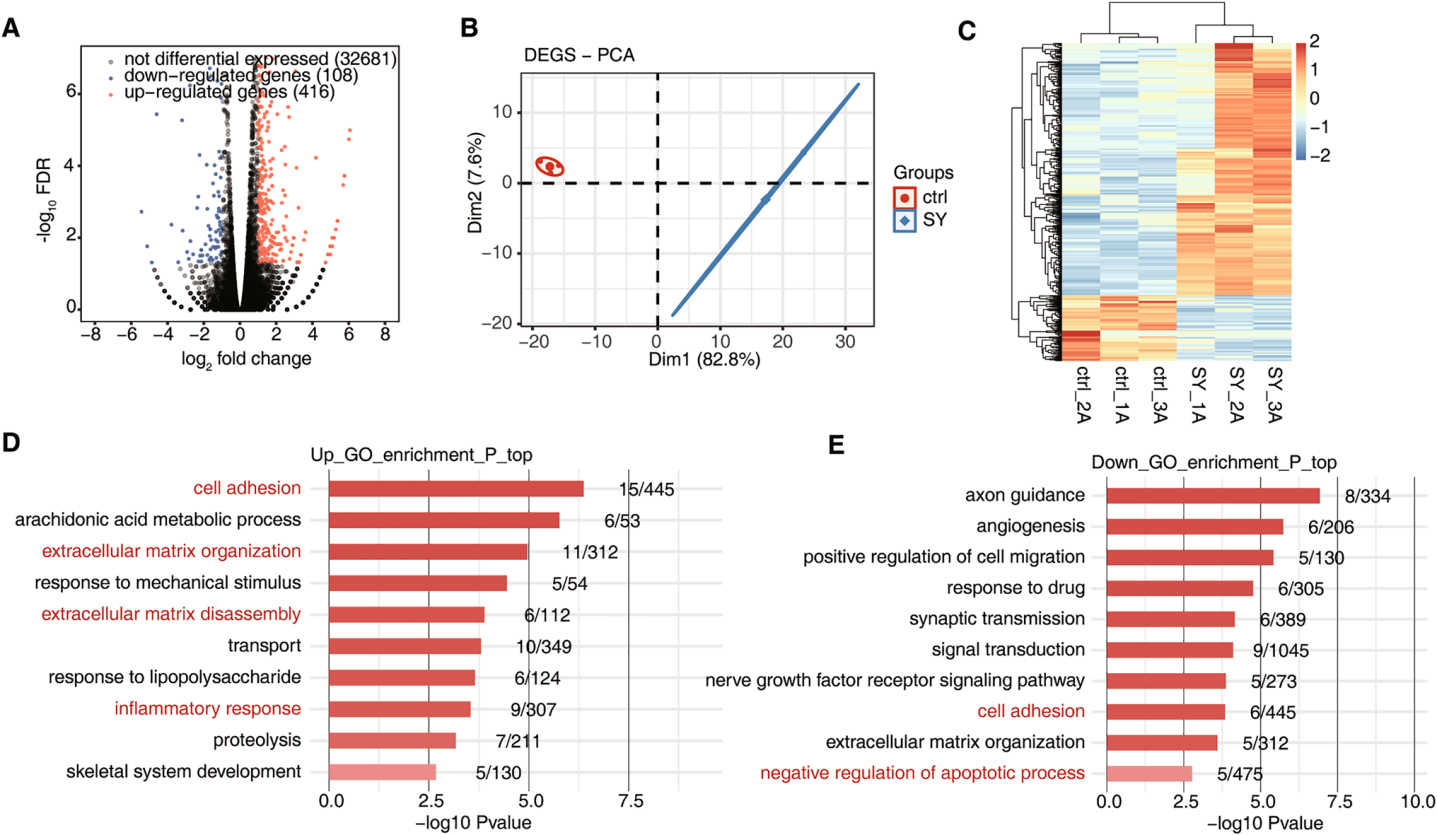
Transcriptome analysis of differentially expressed genes (DEGs) between renal tubular epithelial cells in which sodium oxalate crystallization was induced and control cells. **A** Volcano plots showing DEGs between SY and Ctrl samples. A false discovery rate (FDR) ≤ 0.05 and a fold change (FC) ≥ 2 or ≤ 0.5 were the criteria for DEG identification. **B** Principal component analysis (PCA) of SY and control (Ctrl) samples in human renal tubular epithelial cells was based on the fragments per kilobase of transcript per million reads (FPKM) values of all DEGs. The ellipse of each group is the confidence ellipse. **C** Heat map of 3SY and 3Ctrl samples based on all DEG FPKM values. **D**, **E**. Gene Ontology (GO) analysis of DEGs categorized into upregulated genes (**D**) and downregulated genes (**E**)

**Fig. 2:**
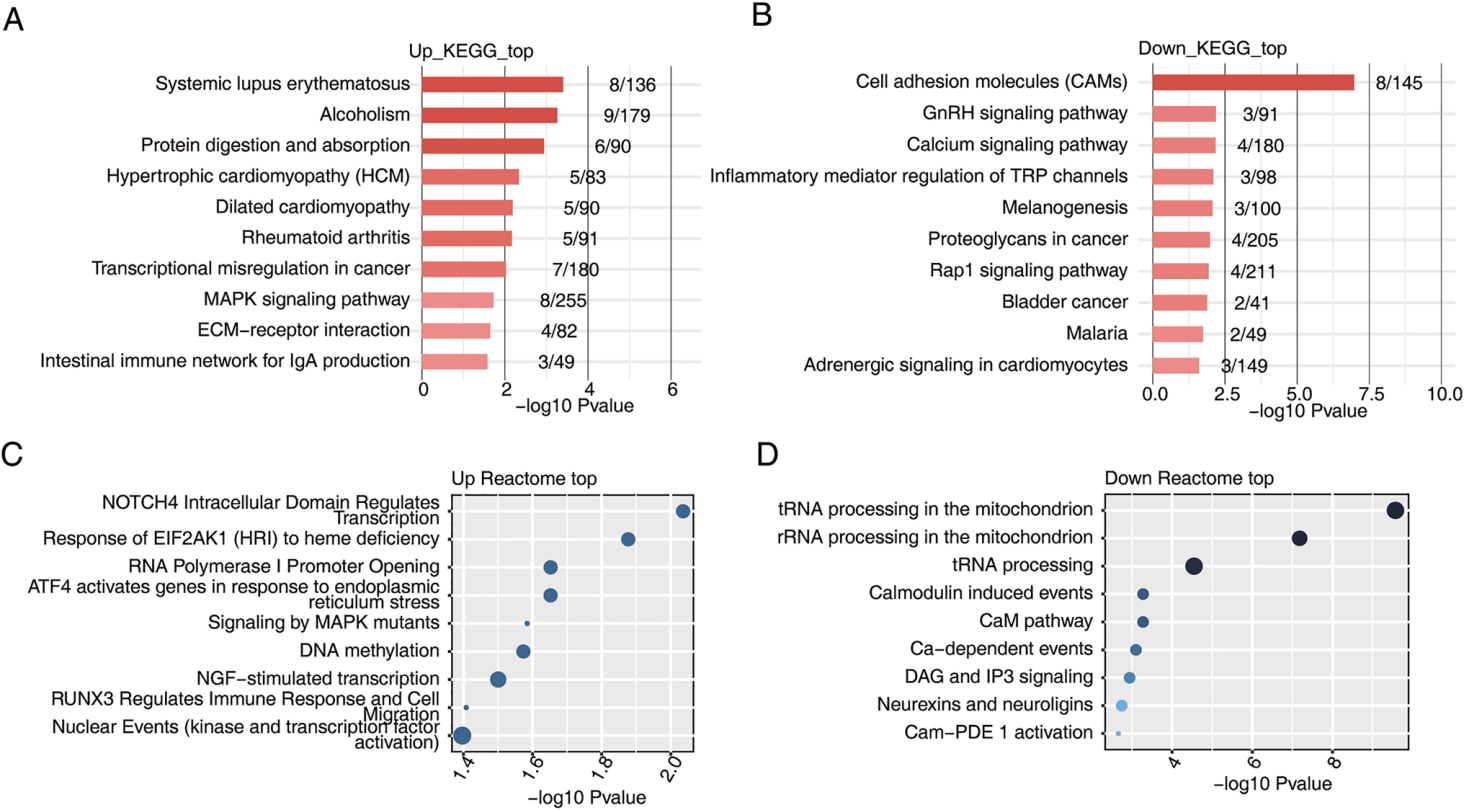
Functional pathway analysis of differentially expressed genes (DEGs) in SY and Ctrl samples of human renal tubular epithelial cells. **A**, **B** Kyoto Encyclopedia of Genes and Genomes (KEGG) analysis of the DEGs categorized into up- and downregulated genes. **C**, **D** Reactome analysis of DEGs categorized into up- and downregulated genes

A total of 33,205 genes were identified, of which 416 were significantly upregulated and 108 were downregulated (Fig. 1A). The results of GO and KEGG enrichment analyses revealed that most DEGs were associated with cell adhesion, inflammatory responses, inflammatory mediator (e.g., arachidonic acid) secretion and other processes (Figs. 1 and 2) as well as with human autoimmune diseases such as systemic lupus erythematosus and rheumatoid arthritis. The cell adhesion pathway, which was significantly associated with upregulated genes, was the focus of our attention. In previous studies, cell crystal adhesion was found to play an important role in the development of kidney stones, acting as a key factor in the stone formation process. Upregulated genes were mostly involved in cellular activities such as the regulation of intracellular factor receptors, activation of RNA enzymes, and methylation of DNA (Fig. 2C). These genes might have a regulatory role at the cellular transcriptional level in promoting the deposition of kidney crystals.

Genes associated with the negative regulation of apoptosis and cell adhesion pathways were downregulated, indicating decreased expression of genes associated with apoptosis inhibition. These events might be associated with the regulation of renal tubular epithelial injury. Apoptosis, as a type of programmed death, enables the body to clear damaged or senescent cells without inducing an acute inflammatory response. Accordingly, downregulation of genes involved in local negative apoptosis regulation may amplify the inflammatory response and the deposition of crystals. Furthermore, these downregulated genes may significantly affect the expression of cell adhesion molecules (CAMs) and the processing of mitochondrial tRNAs and rRNAs (Fig. 2D). These observations are in line with previous results that showed changes in cellular function during crystal deposition and oxidative damage to mitochondria.

### RT-qPCR experiments revealed the accuracy and consistency of the sequencing results

**Fig. 3:**
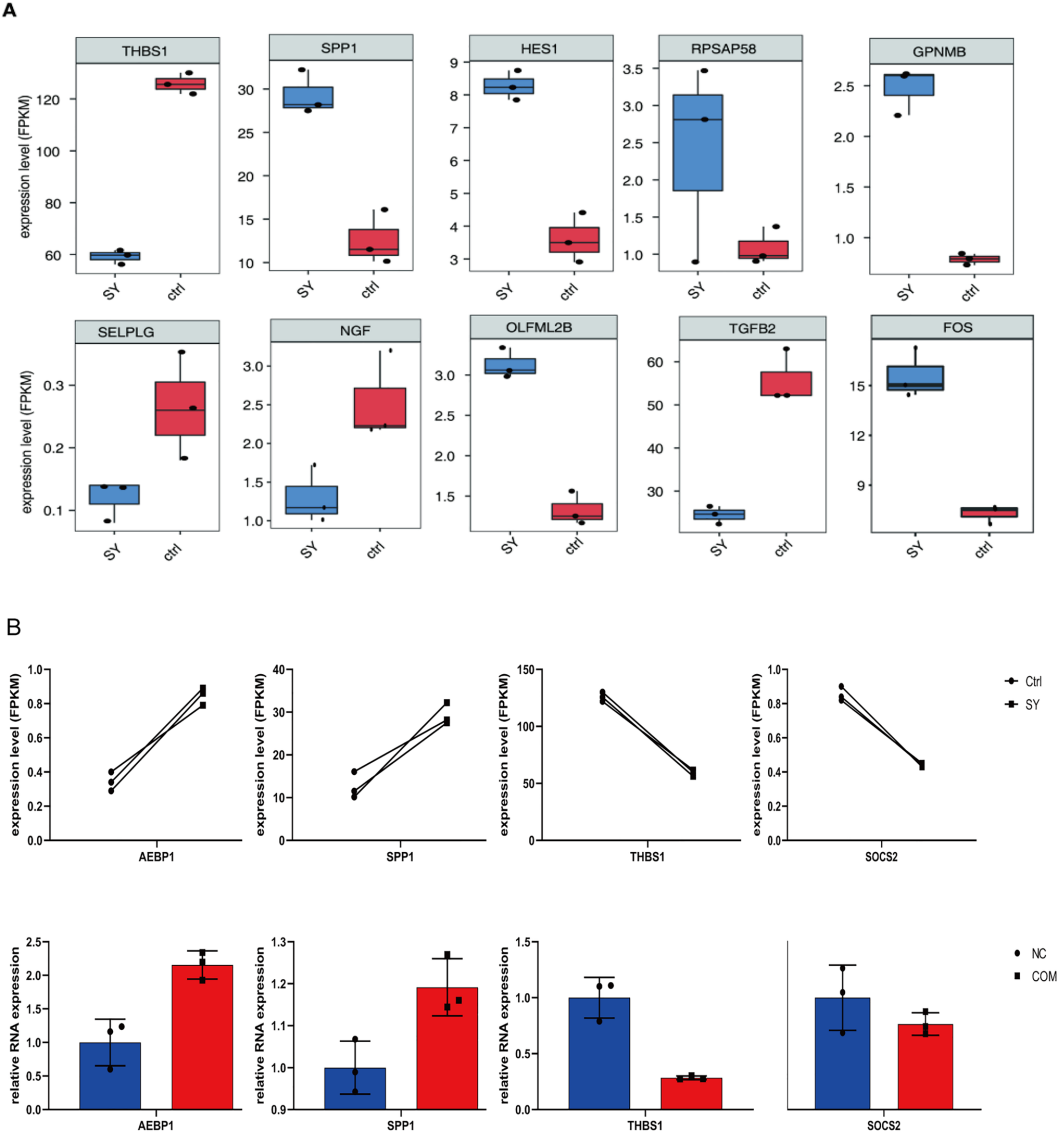
Validation of the expression of important genes involved in nephrolithiasis. **A** Box plot showing the expression levels of differentially expressed genes (DEGs) involved in cell adhesion, extracellular matrix organization, extracellular matrix disassembly, inflammatory responses, and negative regulation of apoptosis, as indicated by term annotation analysis. **B** Scatter plot showing the results of RNA sequencing and RT-qPCR as the means ± standard deviations, with indicated statistically significant differences between groups (*p* < 0.05)

For validation of the results, we randomly selected two genes that were significantly differentially expressed. Real-time fluorometric qPCR was used to amplify and obtain CT values, and the relative expression of RNA was calculated by the -ΔΔCT value-correction method. The RT-qPCR results were found to be in general agreement with the sequencing results (Fig. 3B). The results from the experiments showed that differences in gene expression were reasonably consistent and provided a basis for further research.

### Identification of many alternative splicing events

**Fig. 4:**
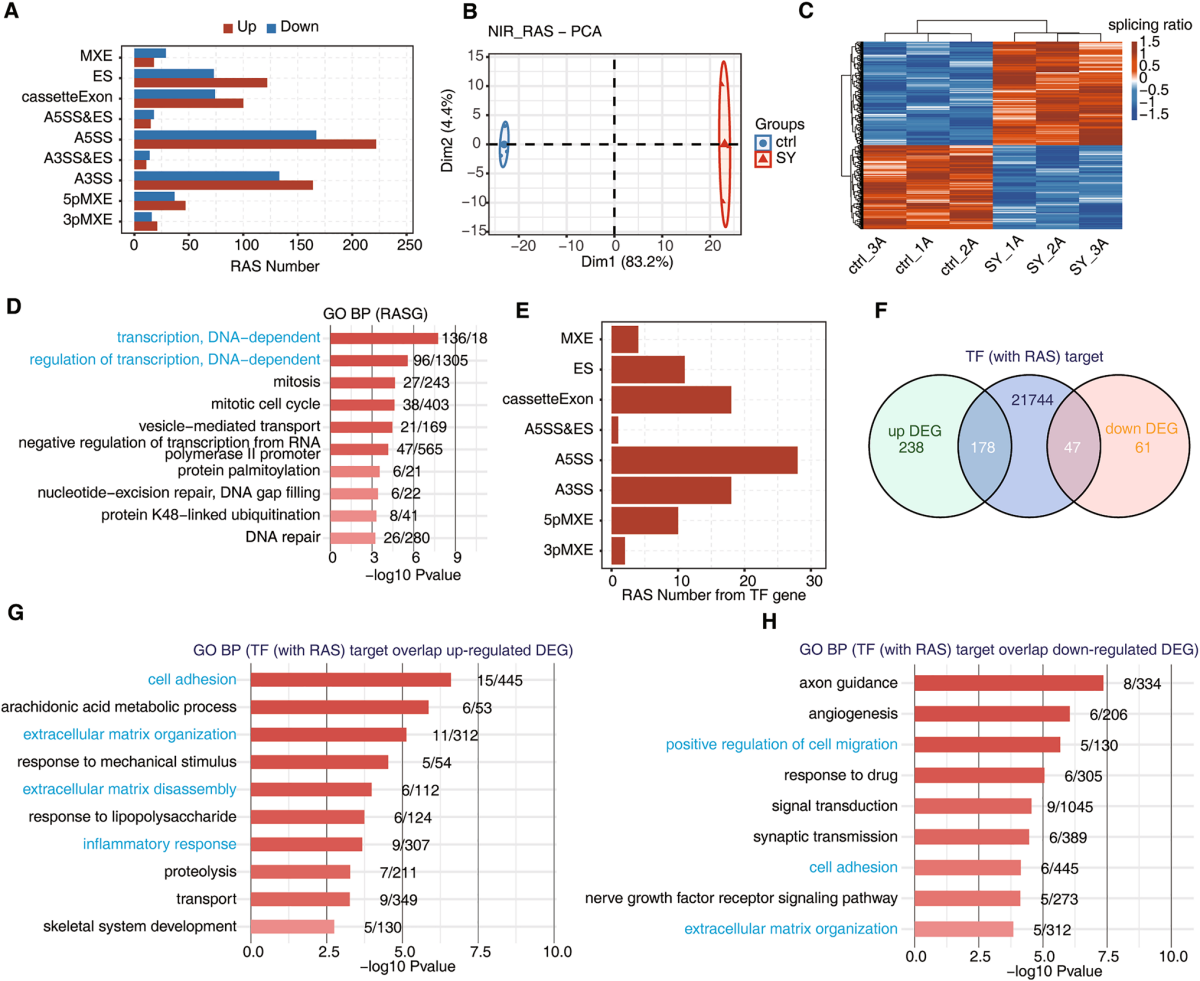
Global features and enriched functions of deregulated alternative splicing events between treated and control samples. **A** Classification of all regulated alternative splicing events (RASEs). X-axis: Number of RASEs. **B** Principal component analysis (PCA) of SY and control (Ctrl) human renal tubular epithelial cells based on the percentage of spliced (PSI) values of all nonintron-retained (NIR) splice events. The ellipse for each group is the confidence ellipse. **C** PSI heat map of all NIR RASEs in SY samples compared to Ctrl samples. The filtration criteria used to identify an ASE were detectable splice junctions in all samples and at least 80% of the samples having 10 or more splice junction reads. **D** Gene ontology (GO) analysis of regulatory alternatively spliced genes (RASGs) in SY samples compared to Ctrl samples. **E** Classification of RASEs in transcription factors (TFs). *X*-axis: number of RASEs. **F** Venn diagram of TF targets with regulated alternative splicing and up- and downregulated genes. TF targets were identified using the Ensembl database and TRRUST database (https://www.grnpedia.org/trrust/). **G** Top 10 GO biological process terms most enriched by upregulated DEGs overlapping with TF (with RAS) target genes. **H** Top 10 GO biological process terms most enriched by downregulated DEGs overlapping with TF (with RAS) gene targets

**Fig. 5:**
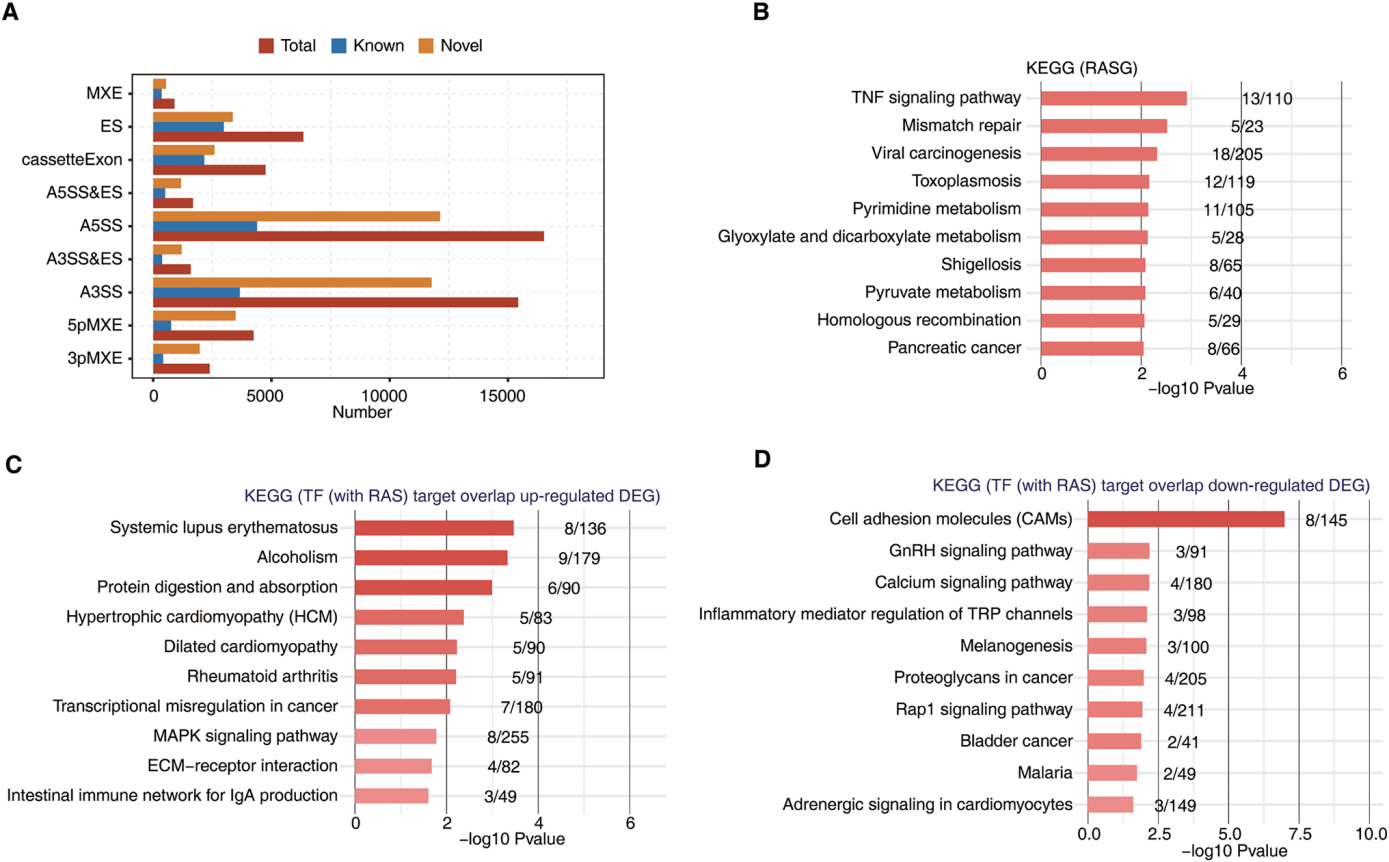
Global features and enriched functional terms associated with dysregulated alternative splicing events (ASEs) between treated and control samples. **A** Bar plot showing the number of known and novel detected ASEs, which were classified into 9 types. **B** Kyoto Encyclopedia of Genes and Genomes (KEGG) analysis of regulatory alternatively spliced genes (RASGs) in SY samples and control (Ctrl) samples. **C** Top 10 KEGG terms most enriched by upregulated DEGs overlapping with TF (with RAS) target genes. **D** Top 10 KEGG terms most enriched by downregulated DEGs overlapping with TF (with RAS) target genes

We identified many differential ASEs. These differential ASEs were analyzed to determine how effectively they distinguished the treatment group from the control group (Fig. 4B, C). Among the nine differential splicing events that we evaluated, ASEs were most frequent for A5SSs and A3SSs (Fig. 5), and among the DEGs, most ASEs involved ES (exon skipping), an A5SS (alternative 5' splice site) or an A3SS (alternative 3' splice site) (Fig. 4A). Genes that underwent selective splicing were most commonly enriched in transcriptional regulation, mismatch repair and related pathways.

Target genes of transcription factors (TFs) that undergo differential AS were extracted from the Ensembl database and Transcriptional Regulatory Relationships Unraveled by Sentence-based Text mining (TRRUST) database. Then, the target genes that overlapped with DEGs were identified, and many of these DEGs were found to be potentially regulated by the assessed TFs (Fig. 4E, F).

### TF (with RAS) targets that overlap DEGs in functional enrichment analysis

We selected a set of genes from the larger group of genes with expression affected by TFs in which variable splicing events had been identified. We analyzed this gene set, TF gene targets that had undergone RASEs and DEGs by generating a Venn diagram. We found many intersecting genes, suggesting that these variable splicing events influenced the differential expression of related genes (Fig. 4F). GO and KEGG analyses revealed that these overlapping genes regulated by variable splicing events were highly enriched in cell adhesion, cellular response, inflammatory response, cell migration and other pathways associated with renal calculi.

**Fig. 6:**
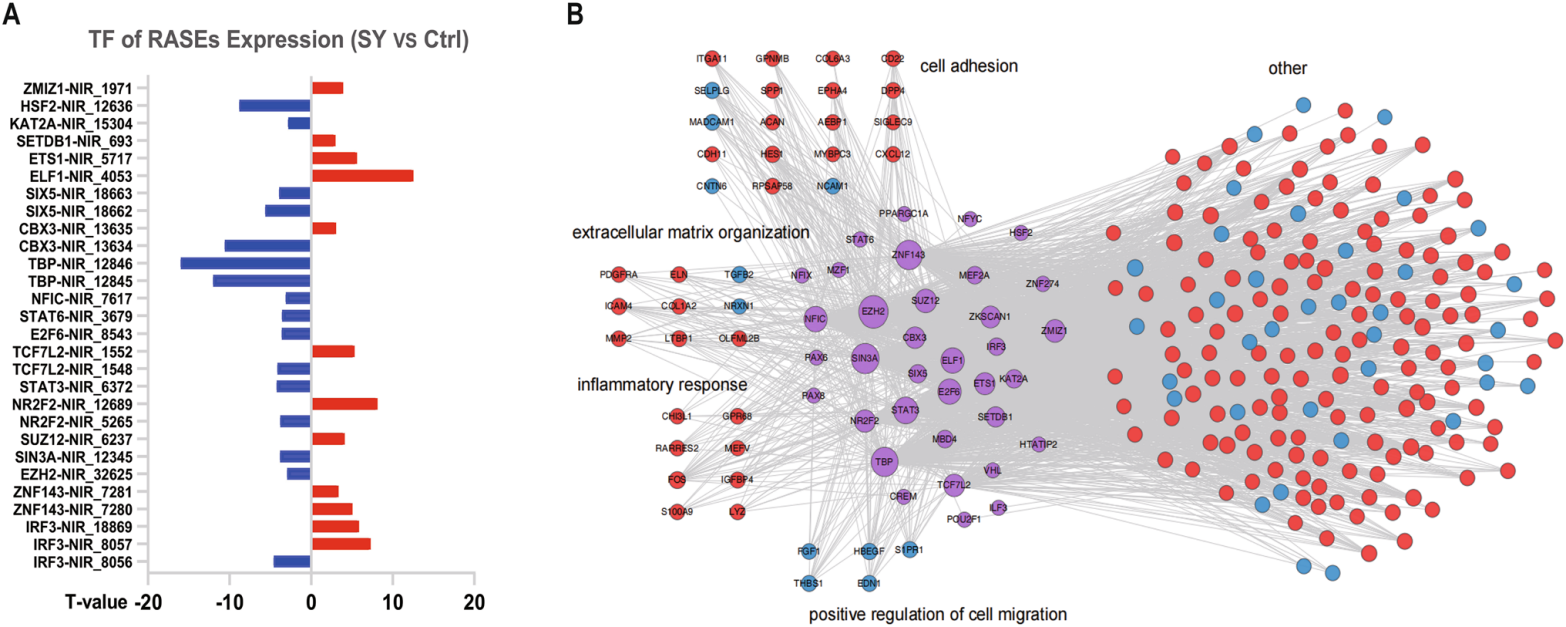
Transcription factor–differentially expressed gene (TF–DEG) network comprising TFs (middle) and DEGs. **A** Differences in the expression of different spliceosomes of TFs with regulated alternative splicing. **B** TFs were significantly associated with cell adhesion, inflammatory responses, extracellular matrix organization, positive regulation of cell migration and other processes. Differences in spliceosomes are expressed as *T* values, and *P* < 0.05 was considered significant for the experimental group compared with the control group. Only TF–DEG connections found in the Ensemble or TTRUST database were included in the network. DEGs were classified according to Gene Ontology (GO) terms. Red circles indicate upregulated genes, and blue circles indicate downregulated genes

Furthermore, we established the regulatory network of TFs that undergo variable splicing and mediate DEG expression (Fig. 6). Additionally, we identified and demonstrated the involvement of important TFs in the regulatory network (for instance, IRF3, STAT family members, TBP, ZNF143, and NR2F2) with DEGs in the regulatory processes of cell adhesion, inflammatory response, extracellular matrix composition, cell migration, etc. Pathways involved in cell adhesion (CD22, SPP1, AEBP1, etc.) and the inflammatory response (GPR68, S100A9, FOS, etc.) were significantly upregulated, while those involved in cell migration-related genes (THBS1) were significantly downregulated, suggesting an altered functional state of the cells.

## Discussion

ASEs exert important effects on the regulation of gene expression and contribute to the diversification of gene expression and protein properties. ASEs have also been a hot spot in the field of RNA-seq analysis and drug development [14, 15]. Studies on renal ASEs have been reported, for instance, by Jing et al., regarding the involvement of alternative splicing of circular RNAs (circRNAs) in the regulation of renal cancer specificity [16]. Wineberg et al. studied the regulatory role of mRNA AS at the single-cell level in kidney development and differentiation [17]. However, few ASEs have been reported in studies on urinary stones, which characterize a major kidney disease. Our study aimed to fill this gap.

In previous studies on the effects of crystal deposition on gene expression in renal tissue, Khan et al. used the ethylene glycol (EG) model and found that crystal retention may cause genetic alterations in inflammatory factors [18]. Koul et al. showed that HK-2 cells undergo compositional changes and altered biological processes when stimulated with different oxalates [19]. Recently, a data set from Randall's plaque also showed that CaOx causes pro-oxidative and pro-damage gene expression alterations [20]. We believe that the differences in our data are due to the different choices of mold-making methods. We innovatively used nanocrystals formulated from clinical stones to stimulate renal tubular epithelial cells and investigated the effects of this stimulation on genomic changes and selective splicing events. Interestingly, we found that crystal deposition can cause changes in renal tubular epithelial adhesion processes as well as inflammatory damage to renal tissue, which is consistent with the previous studies.

Based on RASE analysis and functional clustering analysis of associated genes, we established a network map showing the regulation of gene expression by ASEs and found that AS genes are highly concentrated in transcriptional regulatory pathways. In stone formation, ASEs in TFs can regulate cell adhesion, apoptosis (particularly affecting negative regulation), and tissue inflammatory responses. Crystallographic cell adhesion remains an important process in the development of renal calculi [2, 5, 21]. Crystallization stimulation may induce an immune response in renal tubular epithelial cells and affect changes in cellular functions [22]. Among these changes, oxidative damage has been reported most frequently. Increased intracellular generation of reactive oxygen species (ROS) can induce changes in gene expression and even lead to DNA damage [23]. Oxidative stress may be the cause of increased ASEs. The transcription factors identified herein, such as IRF3, STAT3, TBP, SIN3A, and PAX8, have been reported to be involved in transcriptional regulation [24–28]. For example, STAT3 and IRF3 can affect human immune processes and the cell cycle through transcriptional regulation [24, 29].

In injury caused by renal stone crystal deposition, we reported that AEBP1, SPP1, THBS1, ITGA11, and SOCS2 may be involved in transcriptional regulation. Based on previous studies, we determined that injury caused by crystal deposition is frequently accompanied by renal fibrosis and that THBS1, ITGA11, and SOCS2 can affect the fibrotic process [30–32]. Moreover, Murphy et al. found that THBS1 promoted tissue fibrosis by activation of the TGF-B pathway [33]. We were particularly intrigued by the study by Cao's team, which showed that THBS1 increased cell adhesion and migration via the YAK/FAK signaling pathway [34]. The downregulation of THBS1 expression was inferred as a possible protective factor against injury by renal stone crystal deposition, protecting the renal tubular epithelium from crystal damage, promoting fiber repair and reducing the cell adhesion capacity. However, further experiments are needed to confirm this inference. In contrast, Liu et al. found that AEBP1 activated the NF-kB pathway and affected the growth state of cells [35, 36]. In addition, Deng, Wang et al. found that SPP1 promoted cell proliferation and migration through the NF-kB pathway [37, 38]. Overall, all identified genes regulated by ASEs affect cell adhesion, damage repair, inflammatory signaling activation and other cellular activities associated with kidney stones.

In the TF–DEG regulatory network, CD22 was differentially expressed. CD22 is a cell-surface B cell-specific receptor involved in cell adhesion and signaling processes [39]. In the development of kidney stones, CD22 may be involved in recruiting leukocytes and triggering the inflammatory response [40]. This study is the first to describe CD22 involvement in kidney stone disease. Previously described adhesion molecules in renal stone disease include CD44, HA, and OPN [21, 41]. In our study, the involvement of CD22 might be related to leukocyte chemotaxis and in vivo clearance of crystals, which are possibilities that need to be further investigated.

In summary, our report of selective splicing events in kidney stone formation fills a gap in the field of AS in basic research on kidney stones. The analytical results provide new insights into the mechanism of urinary stone formation and provide a basis for subsequent research on the specific regulatory mechanism(s) of ASEs and kidney stone formation.
